# Daisaikoto Prevents Post-dieting Weight Regain by Reversing Dysbiosis and Reducing Serum Corticosterone in Mice

**DOI:** 10.3389/fphys.2019.01483

**Published:** 2019-12-13

**Authors:** Takanori Kawashima, Misaki Ogata, Nina Fujita, Ryuji Takahashi

**Affiliations:** Kampo Research Laboratories, Kracie Pharma, Ltd., Tokyo, Japan

**Keywords:** Bacteroidetes, Firmicutes, corticosterone, daisaikoto, dieting, dysbiosis, obesity, weight regain

## Abstract

Weight loss is often temporary and is generally followed by recurrent weight gain and a relapse of metabolic complications, whose severity may be even greater upon recurrence. Preventing recurrent obesity, understanding the control of the energy balance subsequent to weight loss, and reversing the predisposition to obesity are critical factors that warrant an in-depth study. Several Kampo medicines, including daisaikoto, have traditionally been used to manage obesity, but their mechanisms of action are not well studied and their effects on weight regain are unknown. Here, we investigated the therapeutic potential and mechanism of action of daisaikoto in a mouse model of recurrent obesity. The mouse model was established by feeding mice a high-fat diet, followed by a normal chow, and a second course of the high-fat diet. Daisaikoto inhibited not only obesity and regaining of weight post-dieting, but also dysbiosis, thereby overcoming the predisposition to obesity. Furthermore, we found that recurrent obesity or long-term consumption of the high-fat diet elevated serum glucose, insulin, and corticosterone levels, and that daisaikoto lowered serum cholesterol and free fatty acid levels. These results are consistent with the hypothesis that this medication may inhibit lipid absorption by inhibiting pancreatic lipase. However, daisaikoto had no effect on the body weight of lean mice fed a normal chow, suggesting that although this medicine prevents lipid absorption, it does not cause excessive weight loss. In conclusion, our results elucidate the mechanisms underlying daisaikoto activity, and suggest that it may serve as a safe and effective anti-obesity drug.

## Introduction

Obesity, a major risk factor for metabolic syndrome, leads to morbidities such as type 2 diabetes, dyslipidemia, cancer, mood disorders, heart disease, reproductive disorders, liver disease, and hypertension, and can thus markedly reduce the quality of life (Kyrou et al., 2000). Obesity is characterized by accumulation of excess body fat, generally due to a high calorie intake accompanied by low calorie consumption. However, obesity is a complex condition and is not fully reversed simply by calorie restriction. Thus, obesity has been linked not only to eating habits, drinking, and exercise, but also to factors such as sleep duration (Watanabe et al., 2010; Itani et al., 2011; Kobayashi et al., 2012), smoking (Bamia et al., 2004; Chiolero et al., 2007), psychosocial and socioeconomic factors (Ishizaki et al., 2008; Toyoshima et al., 2009), work conditions (Solovieva et al., 2013), sex hormones (Lovejoy et al., 2009), aging (Kitamura et al., 2014), and the gut microbiome.

Although several candidate anti-obesity drugs have been developed, they often show insufficient pharmacological effects or cause side effects, preventing their widespread use (Caveney et al., 2011). Moreover, when drug-induced weight loss is achieved, it is typically followed by recurrent weight gain and a relapse of metabolic derangements, whose severity may be even more pronounced after the weight loss (Anastasiou et al., 2015). Reduced energy intake is counteracted, in the short term, by mechanisms that slow down the metabolism and stimulate calorie intake, ensuring that the initially lost weight is eventually regained.

Notably, the gut microbiome has been linked to weight regain because it affects host energy homeostasis via digestion, metabolism of bile acids, and production of endotoxins. In particular, the gut microbiota associated with obesity is believed to be less diverse than that in non-obese individuals, with a reduction in Bacteroidetes and an increase in Firmicutes (Le Chatelier et al., 2013; Million et al., 2013). Such microbiomes are believed to be functionally abnormal and able to extract energy from the gastrointestinal contents with a high efficiency, ultimately leading to negligible weight loss or even fat accumulation (Holmes et al., 2012). Accordingly, modification of the gut microbiota through probiotic and prebiotic interventions and fecal transplants has been attempted with the aim of reversing obesity (Vrieze et al., 2012; Chambers et al., 2015). Similarly, bariatric surgery apparently induces changes in the intestinal microbiota, which contribute to the maintenance of an optimally low weight (Vrieze et al., 2012). Of particular relevance to this study, high-fat dieting is known to induce constipation (Taba Taba Vakili et al., 2015), which affects the speed of movement of the gastrointestinal content and the composition of the intestinal bacterial microbiota (Murillo-Rincon et al., 2017), and some evidence indicates that metabolic complications associated with recurrent weight gain might be due to a persistent post-obesity microbiome (Thaiss et al., 2016). We were therefore motivated to investigate whether metabolic disorders due to periodic consumption of a high-fat diet are associated with dysbiosis.

Stress is believed to be an additional factor that promotes weight regain (Elfhag and Rössner, 2005). For example, a 1998 study demonstrated that elevated levels of stress could promote the secretion of cortisol, which in turn stimulated the production of serum glucose and insulin (Rosmond et al., 1998). High-dose cortisol has been found to be linked to increases in serum insulin, even in healthy volunteers (Whitworth et al., 1994). Similarly, administration of high doses of insulin to adults with type 1 diabetes resulted in excessive weight gain compared with that observed with conventional therapy (The Diabetes Control and Complications Trial Research Group, 2001). Moreover, long-term high-fat dieting can lead to elevated levels of serum corticosterone (Shen et al., 2017; Lizarbe et al., 2019).

The foregoing observations highlight the necessity of preventing recurrent obesity, understanding control of the energy balance after weight loss (Benton and Young, 2017), and reversing the predisposition to obesity. In this regard, several Kampo medicines, including daisaikoto, which is listed in the Chinese medicinal texts Shanghan Lun and Jingui Yaolüe, have been found to be beneficial for managing obesity, and are rapidly metabolized in the blood (Wakui et al., 1992; Takeda et al., 1995; Li et al., 2013, 2017; Kwon et al., 2018; Zhao et al., 2018). Because many Chinese medicines, including daisaikoto, are composed of multiple crude drugs and ingredients, it is difficult to determine which of the ingredients is responsible for the medicinal effect. Daisaikoto reportedly inhibits pancreatic lipase activity, lowers serum triglycerides (Matsuo et al., 2018), stimulates glucose uptake, modulates insulin signaling (Kuo et al., 2019) and plasma lipid levels, and alleviates atherosclerotic lesions (Yoshie et al., 2004). Although multiple mechanisms of action of daisaikoto against obesity have been proposed, its effects on weight regain are yet to be determined. In this study, we investigated the therapeutic potential of daisaikoto, a formulation used in the traditional Kampo medicine system, in a mouse model of recurrent obesity.

## Materials and Methods

### Plant Materials

Daisaikoto was produced by Kracie Pharma (Toyama, Japan) as a powder of dried *Bupleurum* root (6 g), *Pinellia* tuber (4 g), *Scutellaria* root (3 g), peony root (3 g), jujube fruit (3 g), immature orange fruit (2 g), ginger rhizome (1 g), and rhubarb (1 g). These plants were identified based on their external morphology and authenticated based on marker compounds, according to the Japanese Pharmacopoeia, as well as using our company’s standards. The powder (lot no. 16042202) was mixed at 3% (w/w) with a normal chow or high-fat diet as needed.

### High-Performance Liquid Chromatography

Daisaikoto powder was extracted with 50% methanol with shaking. After centrifugation, the resulting supernatant was analyzed by high-performance liquid chromatography using a Shimadzu LC-20AD system, equipped with an SPD-M20A detector operating at 240–380 nm (Shimadzu, Tokyo, Japan), on a YMC-Pack ProC reversed-phase column (18φ, 2.0 × 150 mm; YMC, Kyoto, Japan), maintained at 20°C. Samples were eluted at 0.2 mL/min using 0.1% formic acid in water (solvent A) and 0.1% formic acid in acetonitrile (solvent B) over a gradient of 5–70% solvent B from 0 to 90 min, 70% solvent B from 90 to 100 min, 70–5% solvent B from 100 to 110 min, and 5% solvent B from 110 to 120 min.

### Reagents and Mouse Diets

A corticosterone (human, rat, and mouse) ELISA kit was purchased from Immuno-Biological Laboratories International (Hamburg, Germany). Triglyceride, cholesterol, and non-esterified free fatty acid assay kits were purchased from Wako (Tokyo, Japan). Mouse serum was diluted following the manufacturers’ instructions. Serum glucose was measured using Stat Strips (Nipro, Osaka, Japan). CE-2 was purchased at the CLEA Japan, Inc. (Tokyo, Japan). It contained 8.65% water, 25.09% crude protein, 4.48% crude fat, 5.15% crude fiber, 6.95% crude ash, and 49.68% available non-nitrogen. HFD-60 was purchased from Oriental Yeast (Tokyo, Japan). It contains 9.00% water, 22.02% crude protein, 33.21% crude fat, 3.06% crude fiber, 6.31% crude ash, and 26.40% useless non-nitrogen.

### Animals

Five-week-old female C57BL/6 mice were purchased from SLC (Shizuoka, Japan) and acclimated for 2 weeks at 24 ± 2°C under a 12-h light/dark cycle (lights on from 08:00 to 20:00), with *ad libitum* access to chow and water. All efforts were made to minimize suffering and the number of animals used (*n* = 5 per group). Experimental protocols were reviewed and approved by the Experimental Animal Care Committee of Kracie Pharma.

### Model of Post-dieting Weight Regain

**FIGURE 1:**
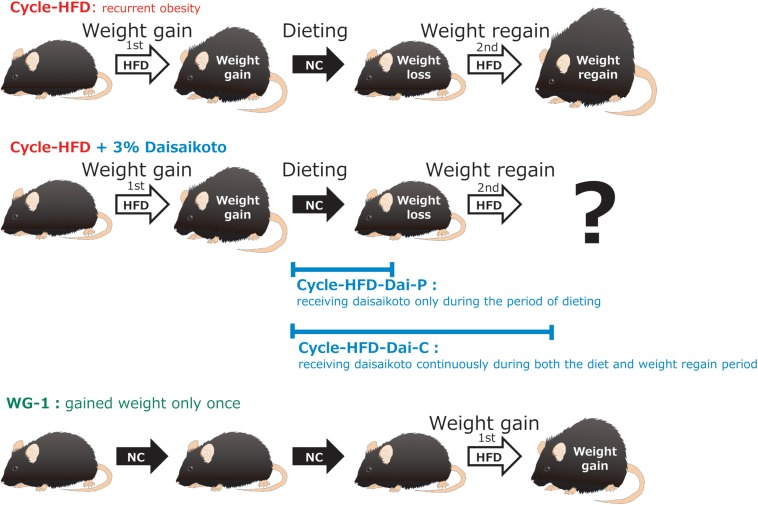
Flow of the experiment. Recurrent obesity was induced through a cycle of feeding a high-fat diet, followed by a normal chow and then the high-fat diet again (Cycle-HFD). A subgroup of the recurrently obese mice was fed a chow that was supplemented with 3% daisaikoto between the cycles of high-fat dieting (Cycle-HFD-Dai-P), whereas the rest were fed the supplemented chow until the end of the experiment (Cycle-HFD-Dai-C). Mice that were continuously fed the normal chow (CE-2) were used as controls, along with the mice that were continuously fed the high-fat diet (HFD-60) and those that only received the high-fat diet in the second cycle (WG-1).

Our mouse model of post-dieting weight regain is equivalent to that used in many other studies, and can be considered to encompass three processes: weight gain, dieting, and weight regain. These have been addressed in many publications, including a study by Thaiss et al. (2016). The length and main features of the three processes examined in this study are summarized below. Mice were first divided into six groups (*n* = 5 per group; Figure 1). (1) Cycle-HFD, which modeled post-dieting weight regain on administration of a high-fat diet, followed by a normal chow, and a second course of the high-fat diet. (2) Similar to group 1, but receiving daisaikoto only during the period of dieting; Cycle-HFD-Dai-P. (3) Similar to group 1, but receiving daisaikoto continuously during both the diet and weight regain period; Cycle-HFD-Dai-C. (4) A group that gained weight only once; WG-1. (5) CE-2, a control group that received a regular diet; CE-2. (6) HFD-60, a control group that received a constant high-fat diet; HFD-60.

#### Weight Gain Process

Mice were initially fed the high-fat diet for 7 weeks until they reached a weight that was significantly higher than that of the mice fed the normal chow (*p* < 0.05).

#### Dieting Process

Obese mice were fed CE-2 instead of HFD-60. There are many studies in which the dieting period was maintained until the weight of the dieting group decreased to that of the normal group. However, in this study, the HFD-60 group reached almost the same weight as the normal group within approximately 2 weeks. Therefore, the duration of the diet was extended, as the weight regain depression effects caused by daisaikoto could not be predicted.

It has been reported that the alteration in the gut microbiome (dysbiosis) caused by the onset of obesity improves after a prolonged period of dieting, but it has also been confirmed that weight regain easily occurs within approximately 3 months of the dieting period in advanced examination. It has also been reported that the duration of changes in the gut microbiome after discontinuation of the administration of Chinese medicine varies according to its dose and duration (Miyoshi et al., 2018). As Chinese medicine often improves its targeted condition within 2–3 months when there are no side effects, we used a dieting period of 3 months. In practice, even shorter treatment durations may be effective.

#### Weight Regain Period

Many studies consider that the weight regain period lasts until the moment when the recurrent obesity group reaches a weight comparable to that of the HFD-60 group. To model post-dieting weight regain, mice were fed the high-fat diet again until their weight became comparable to that of the mice maintained on a continuous high-fat diet (Figure 1).

### Daisaikoto Treatment

Daisaikoto was administered to the recurrently obese mice during the weight loss phase or until a beneficial effect was observed after the return to the high-fat diet (Figure 1). In addition, the effect of daisaikoto supplementation was also investigated in 5-month-old obese C57BL/6 mice that continuously received the high-fat diet.

### Tissue Sampling and Blood Collection

Body weight was measured as an index of obesity, along with food/calorie intake, fat weight, muscle weight, and levels of serum cholesterol, triglycerides, free fatty acids, glucose, and corticosterone. The mice were decapitated, and trunk blood was collected into a microtube containing 10 μL of 100 mg/mL EDTA, followed by centrifugation for 15 min at 1,500 × *g* at 4°C. Plasma was obtained from the blood and stored at −80°C until analysis. The plasma samples were analyzed following the manufacturers’ instructions for each kit. Visceral (mesenteric) fat and the gastrocnemius and soleus muscles were collected and weighed to evaluate their correlations with body weight.

### Analysis of 16S rRNA Gene Sequences in Fecal Samples

Following dissection of the mice, the feces were collected directly from the rectum, placed in sterile containers, and immediately frozen in liquid nitrogen. They were then stored at −80°C until they were used for sequencing bacterial 16S rRNA genes at Takara Bio (Shiga, Japan). The scissors and forceps used for the collection were washed each time and sterilized by fire to prevent contamination of bacteria. Briefly, DNA was extracted using the NucleoSpin microbial DNA kit, and the V3–V4 segment of the bacterial 16S rRNA gene was amplified using the universal primers 314F (5′-TCGTCGGCAGCGTCAGATGTGTATAAGAGACAGCCTACG GGNGGCWGCAG-3′) and 806R (5′-GTCTCGTGGGCTC GGAGATGTGTATAAGAGACAGGGACTACHVGGGTWTCT AAT-3′), which were modified with Illumina adaptor overhang sequences. The amplicons were processed using a 16S (V3–V4) metagenomic library construction kit for NGS, according to the manufacturer’s protocol. The libraries were then sequenced using an Illumina MiSeq system with 250-bp paired-end reads. Subsequently, the reads were processed using Quantitative Insights Into Microbial Ecology^1^, as described in previous studies (Caporaso et al., 2010; Thaiss et al., 2014). Briefly, reads compiled in quality-controlled FASTA files were split into barcoded samples, classified using RDP Classifier^2^, and tabulated as operational taxonomic units, which were then mapped using Greengenes. Rarefaction, an index of bacterial diversity, was used to exclude samples with insufficient reads. Adaptor sequences, pyrosequencing errors, and chimeras were removed, and sequences were clustered into operational taxonomic units at 97% identity using CD-HIT-OTU.

### Statistical Analysis

All statistical analyses were performed with EZR (Saitama Medical Center, Jichi Medical University, Saitama, Japan), which is a graphical user interface for R (R Foundation for Statistical Computing, Vienna, Austria 2.3-0). All data are expressed as the mean ± standard error of the mean (SEM). Statistical comparisons were performed using one-way analysis of variance, followed by Dunnett’s test or Tukey’s test, and Steel’s test was used for data for which the *P*-value of the Bartlett test was less than 0.01. Differences with *p* < 0.05 were considered statistically significant.

## Results

### High-Performance Liquid Chromatography of Daisaikoto Extract

**FIGURE 2:**
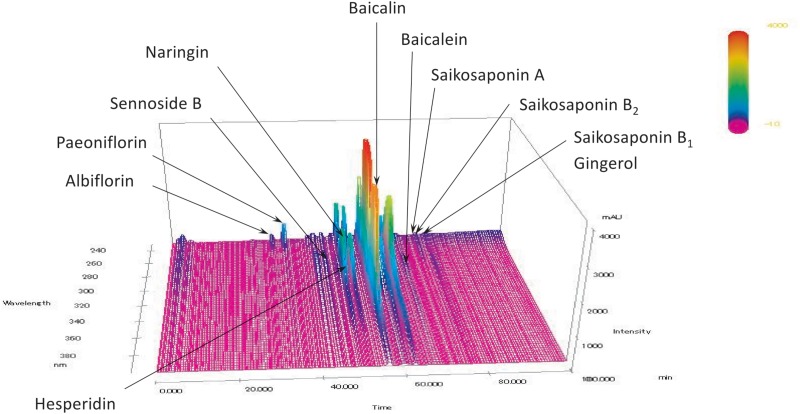
Analysis of daisaikoto components. Albiflorin, Paeoniflorin, Sennoside B, Hesperidin, Baicalin, Baicalein, Saikosaponin A, Saikosaponin B_1_, Saikosaponin B_2_, and Gingerol were identified in the chromatograms based on the comparison of their retention times and UV spectra (240–380 nm) with those of the corresponding reference standards.

The chromatographic profile and composition of daisaikoto are shown in Figure 2. Chemical compounds, including Albiflorin, Paeoniflorin, Sennoside B, Hesperidin, Baicalin, Baicalein, Saikosaponin A, Saikosaponin B_1_, Saikosaponin B_2_, and Gingerol, were identified in the chromatographic profile according to their retention times and UV spectra, based on the corresponding reference standards.

### Model of Recurrent Obesity

**FIGURE 3:**
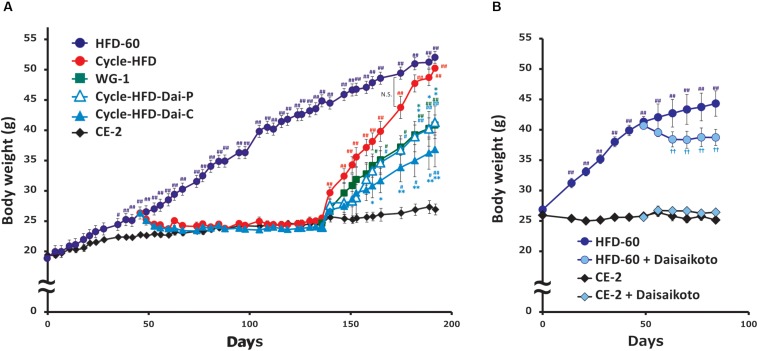
Post-dieting weight regain. **(A)** Recurrent weight gain in mice treated with normal diet and HFD with or without daisaikoto treatment. **(B)** Effects of daisaikoto on obese 5-month-old female C57BL/6 mice on the high-fat diet and lean mice on the normal chow. Data represent the mean ± SEM (*n* = 5). ^#^*p* < 0.05, ^##^*p* < 0.01 vs. normal chow; ^∗^*p* < 0.05, ^∗∗^*p* < 0.01 vs. cycle-HFD; ^††^*p* < 0.01 vs. HFD, as determined by Dunnett’s test.

The effects and mechanisms of action of daisaikoto were investigated in a mouse model that is widely used to study post-dieting weight regain (Figure 1). These mice were observed to be significantly heavier than the control mice after 40 days of the initial cycle of high-fat dieting (Figure 3). Notably, the recurrently obese mice were found to gain more weight in the second phase of high-fat dieting than the mice that only received the high-fat diet during the second cycle. Significant differences in weight, compared with the mice fed a continuous diet of the normal chow, were only observed for 4 days into the second phase of feeding the high-fat diet. In contrast, the mice that only received the high-fat diet during the second cycle became significantly heavier than the control mice after 27 days.

### Effect of Daisaikoto on Recurrent Obesity

Daisaikoto, mixed at 3% (w/w) with the high-fat diet, not only prevented further weight gain in the obese 5-month-old female C57BL/6 mice on the high-fat diet, but induced some weight loss (Figure 3B). However, daisaikoto did not show any discernible effects in the lean mice fed the normal chow (Figure 3B). Furthermore, the mice fed the high-fat diet for 40 days returned to their normal weight within 2 weeks after shifting to the normal chow (Figure 3A). Similarly, supplementation with 3% daisaikoto had no discernible effects during this phase (Figure 3A). Remarkably, the weight gain in these mice was slower in the second phase of high-fat dieting, even when daisaikoto was withdrawn (Figure 3A), and was comparable to that in the mice receiving the high-fat diet only in the second cycle. Continued supplementation with daisaikoto during the second cycle of high-fat dieting further slowed weight gain (Figure 3A).

### Factors Influencing Body Weight

**FIGURE 4:**
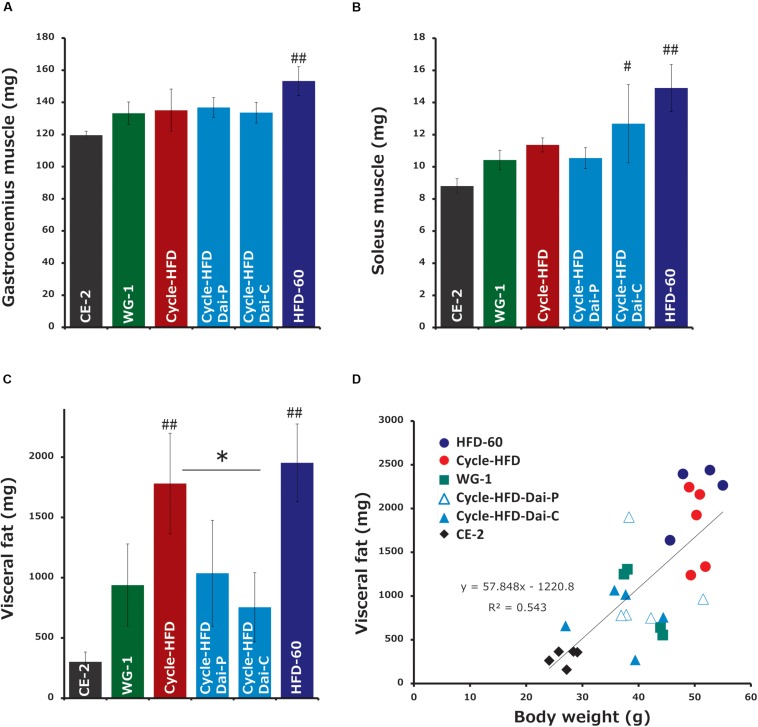
Changes in fat and muscle weights. **(A**,**B)** Muscle weight and **(C)** fat weight. **(D)** Correlation between the visceral fat weight and body weight. Data represent the mean ± SEM (*n* = 5). ^#^*p* < 0.05, ^##^*p* < 0.01 vs. normal chow; ^∗^*p* < 0.05 vs. cycle-HFD, as determined by Tukey’s test.

We found that calorie intake, measured over the week before sacrifice, was correlated with body weight. Notably, the average calorie intake was 21.018 kcal/mouse per day in the mice on the normal chow, 24.288 kcal in the mice only receiving the high-fat diet in the second cycle, 28.032 kcal in the mice undergoing two cycles of high-fat dieting, 22.669 kcal in the mice undergoing two cycles of high-fat dieting and receiving daisaikoto only between the cycles, 22.365 kcal in the mice undergoing two cycles of high-fat dieting and receiving daisaikoto before and during the second cycle, and 34.155 kcal in the mice on a continuous high-fat diet. We predicted that daisaikoto would reduce food intake. However, food intake did not differ between the groups receiving the high-fat diet mixed with daisaikoto and that without daisaikoto after 1 week of daisaikoto administration. Notably, the average calorie intake was 26.019 kcal/mouse per day in the mice on the high-fat diet and 25.869 kcal/mouse per day in the mice on the high-fat diet supplemented with daisaikoto. However, there was a difference (*p* < 0.05) in the body weight between the groups (Figure 3B). And the weights of the gastrocnemius and soleus muscles were comparable among the mice on one or two cycles of high-fat dieting and among those receiving daisaikoto (Figures 4A,B). These results are consistent with low basal metabolic rate and low heat production in the gastrocnemius. Moreover, we found that the visceral fat weight and body weight were strongly and positively correlated (Figure 4C), with a linear correlation coefficient of 0.73 (Figure 4D).

### Serum Factors Associated With Obesity

**FIGURE 5:**
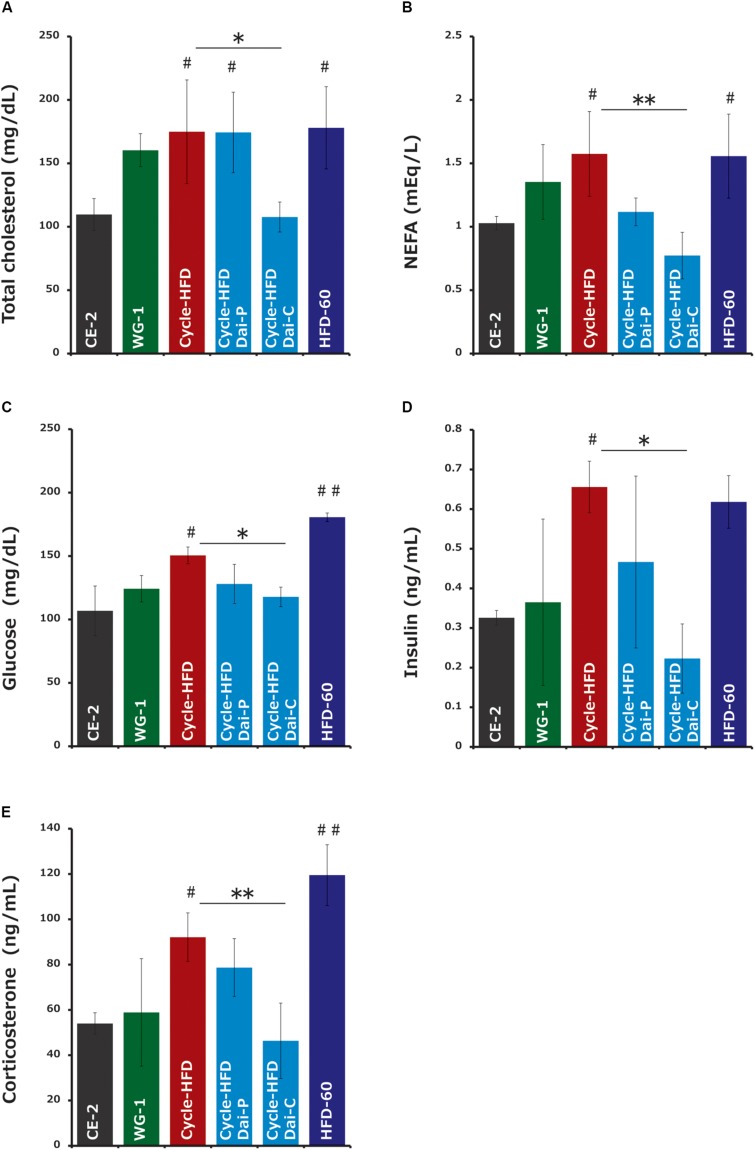
Serum factors associated with obesity and hyperlipidemia. **(A)** Cholesterol, **(B)** free fatty acids, **(C)** blood glucose, **(D)** insulin, and **(E)** corticosterone. Data represent the mean ± SEM (*n* = 4–5). ^#^*p* < 0.05, ^##^*p* < 0.01 vs. normal chow; ^∗^*p* < 0.05; ^∗∗^*p* < 0.01 vs. cycle-HFD, as determined by Tukey’s test in panels **(A,C)** and Steel’s test in panel **(B)**.

Cholesterol, an index of hyperlipidemia, was significantly elevated in the mice undergoing two cycles of high-fat dieting. Supplementation with daisaikoto before and during the second cycle reversed this effect (Figure 5A). Similar trends were observed in the levels of free fatty acids, another index of hyperlipidemia (Figure 5B), as well as in the levels of blood glucose (Figure 5C) and insulin (Figure 5D). Triglyceride levels, also an index of hyperlipidemia, were comparable among all groups (data not shown). Corticosterone, an indicator of stress, was elevated in the recurrently obese mice and in the mice receiving the high-fat diet long-term (Figure 5E). Supplementation with daisaikoto before or during the second cycle of high-fat dieting also significantly reversed this effect.

### Changes in Intestinal Microbiota

**FIGURE 6:**
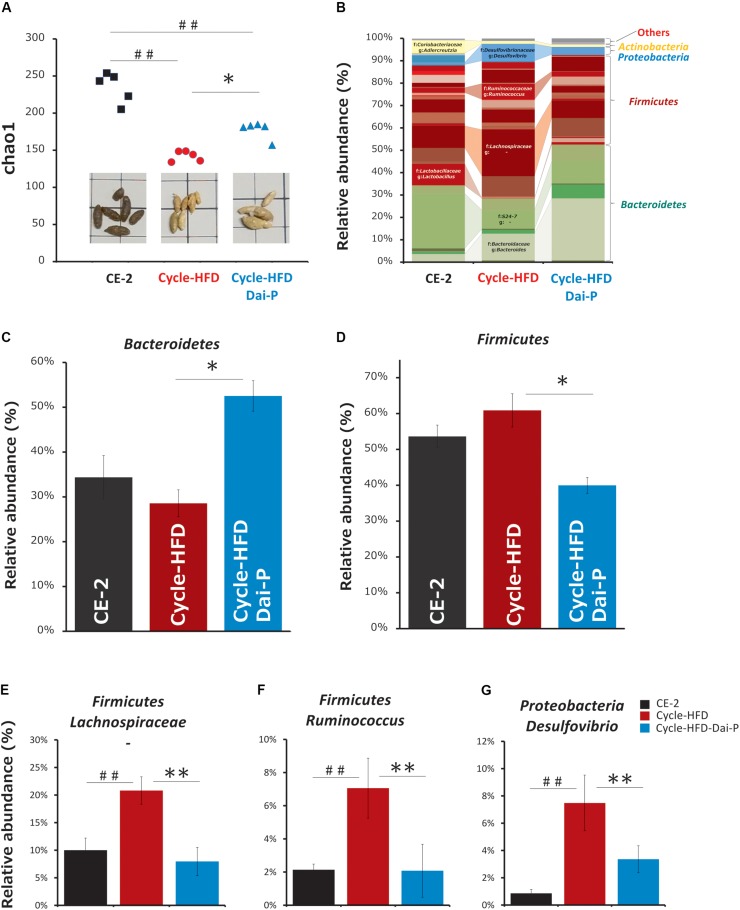
Changes in intestinal microbiota. **(A)** Alpha diversity and **(B–D)** abundance of the intestinal microbiota in fecal samples collected from mice on a normal chow, those receiving a high-fat diet over two cycles (cycle-HFD), and mice receiving the high-fat diet over two cycles with the daisaikoto supplement between the cycles. Daisaikoto inhibited the increase in panel **(E)** an unclassified genus of the family Lachnospiraceae from the order Clostridiales, **(F)** *Ruminococcus*, and **(G)** *Desulfovibrio*. Data represent the mean ± SEM (*n* = 5). ^##^*p* < 0.01 vs. normal chow; ^∗^*p* < 0.05, ^∗∗^*p* < 0.01 vs. cycle-HFD, as determined by Tukey’s test in panels **(A,C,E)** and by Steel’s test in panels **(B,D)**.

Based on the analysis of the fecal 16S rRNA gene sequences, we found that the bacterial alpha diversity was lower – although insignificant – in the mice fed a periodic high-fat diet than in the mice on the normal chow, which was reflected in reduced numbers of Bacteroidetes and increased numbers of Firmicutes in case of mice fed the high-fat diet. Supplementation with daisaikoto reversed these effects, and restored the bacterial alpha diversity by increasing the abundance of Bacteroidetes and diminishing that of Firmicutes (Figures 6A–D). The populations of seven bacterial genera, namely, *Bacteroides*, *Lactobacillus*, *Ruminococcus*, *Desulfovibrio*, *Adlercreutzia*, an unclassified genus of the family S24-7 in the order Bacteroidales, and an unclassified genus of the family Lachnospiraceae in the order Clostridiales, were significantly altered in the recurrently obese mice. However, only *Desulfovibrio*, *Ruminococcus*, and an unclassified genus of the family Lachnospiraceae, showed changes of abundance in the mice fed the daisaikoto-supplemented diet (Figures 6E–G).

## Discussion

Obesity is a major risk factor for metabolic syndrome and is associated with reduced quality of life. However, long-term strategies to attenuate or reverse the obesity epidemic have been ineffective, despite continuous medical and scientific support. Weight loss is often temporary and is generally followed by recurrent weight gain and a relapse of metabolic complications, which may be even more severe than those experienced prior to the weight loss (Anastasiou et al., 2015).

Our investigation of a mouse model of recurrent obesity showed that these mice gained more weight in the second of the two cycles of high-fat dieting than did mice that received the high-fat diet only in the second cycle, suggesting that initial obesity causes a persistent predisposition to obesity and associated morbidities. However, the weight gain was slower in the mice treated with daisaikoto during the weight loss phase and was comparable to the weight gain in the mice that were administered a single course of the high-fat diet. This effect was even more pronounced in the mice treated with daisaikoto during both the weight loss phase and the following cycle of high-fat dieting. Moreover, we found that the visceral fat weight and body weight correlated, indicating that the weight loss with daisaikoto was due to a reduced fat weight.

In a previous study, mice treated with broad-spectrum antibiotics during a weight loss phase were found to gain amounts of weight in the second cycle of high-fat dieting similar to those gained by mice that were only fed the high-fat diet for a single cycle, implying that the metabolic complications associated with recurrent weight gain are due to a persistent post-obesity microbiome (Thaiss et al., 2016). Our data showed that the microbiome in the mice fed a periodic high-fat diet resembled that associated with obesity (Le Chatelier et al., 2013; Million et al., 2013). Of the seven bacterial genera that were significantly altered in these mice, the probiotic genus *Lactobacillus* has previously been reported to attenuate high-fat diet-induced obesity (Tanida et al., 2008). Meanwhile, a loss of Clostridiales is a typical feature of dysbiosis (Sen et al., 2017), and an increase in Ruminococcaceae may promote sugar absorption and fat accumulation (Andoh, 2015). Bacteria of the genus *Desulfovibrio*, whose abundance increases following consumption of a fat-rich diet, secrete inflammatory endotoxins that may trigger metabolic diseases (Zhang-Sun et al., 2015), whereas *Adlercreutzia* species have been found to be enriched in mice fed fish oil (Ziętak et al., 2016).

To study inflammation in the mice fed the high-fat diet and to understand how daisaikoto may be suppressing inflammatory mediators, we performed a supplementary experiment in which 5-week-old ICR (male) mice were fed a high-fat diet for 1 month. Gene expression analysis of inflammatory mediators, including IL-6, TNF-α, and Serpine1 (PAI1), in visceral fat (mesenteric fat) after daisaikoto administration failed to identify any significant changes (*n* = 3). However, the expression of MCP-1, which is responsible for the release of inflammatory cytokines, was significantly decreased (*p* < 0.001) (Supplementary Figure S1; data for the figure has been provided in Supplementary Table S1). In addition, the expression of adiponectin was elevated in these mice. We have previously discussed the relationship between damage (particularly inflammation) caused by fat accumulation and adiponectin (Yokota et al., 2000). Among the inflammatory cytokines secreted by macrophages, adiponectin specifically suppresses only TNF-α expression (Maeda et al., 2002). It has been demonstrated that the expression of TNF-α is increased in the adipose tissue of adiponectin-deficient mice, whereas adiponectin expression is suppressed by TNF-α stimulation in adipocytes. Thus, adiponectin is considered to have an anti-inflammatory effect, as expression levels of adiponectin and CRP mRNAs are inversely correlated in adipose tissue (Ouchi et al., 2003; Supplementary Figure S1).

To our knowledge, this is the first study to find that supplementation with daisaikoto, even temporarily, alleviates dysbiosis and restores bacterial diversity. Various other herbal medicines have also been reported to affect the composition of intestinal microbiota. For example, daikenchuto, which is used to treat adhesive bowel obstruction and “a feeling of coldness in the abdomen,” prevented a loss of microbiome diversity in a rat model of fasting stress (Yoshikawa et al., 2013) in a manner that depended on treatment duration, sex, and treatment dose (Miyoshi et al., 2018). Sennoside, which is present in daisaikoto, is metabolized by the gut microbiota to Rheinanthrone, which promotes peristalsis (Matsumoto et al., 2012), a major driver of microbial diversity. Taken together, the data obtained in the present study imply that daisaikoto prevents not only obesity but also dysbiosis, thereby reducing the predisposition to obesity.

We found that recurrent obesity or long-term consumption of a high-fat diet elevated the serum levels of corticosterone, as previously observed (Shen et al., 2017; Lizarbe et al., 2019), as well as the serum levels of glucose and insulin. Notably, stress promotes weight regain (Elfhag and Rössner, 2005) by elevating cortisol levels, which in turn leads to increases in the serum levels of glucose and insulin (Rosmond et al., 1998). In addition, glucocorticoids, which are classic mediators of stress responses, have been linked to obesogenic eating behaviors, body fat distribution, and metabolism (Cavagnini et al., 2000; Dallman et al., 2003; Adam and Epel, 2007). Corticosterone accumulation, due to dysregulated glucocorticoid signaling, as well as the associated abnormal adipocytokine secretion, induces fat accumulation (Masuzaki et al., 2001). Salivary cortisol is also significantly associated with resistance to weight loss in obese patients (Himeno et al., 2012). Importantly, the effects of recurrent obesity on serum corticosterone, glucose, and insulin were reversed by daisaikoto, indicating that this medicine may prevent weight regain by lowering serum corticosterone.

Finally, we found that daisaikoto lowered the serum levels of cholesterol and free fatty acids, consistent with the hypothesis that this medicine may inhibit fat absorption by inhibiting pancreatic lipase (Matsuo et al., 2018). However, daisaikoto had no effect on the body weight of lean mice, fed a normal chow, which indicates that while this medicine prevents fat absorption, it does not cause excessive weight loss.

This study had some limitations. First, we considered stress due to environmental factors, without performing individual breeding or changing the combination of mice during the experimental period. Hence, it was not possible to examine the extent of exercise, food intake, and metabolism of each individual mouse. Second, we did not collect feces in each phase to examine changes in intestinal microbiota. Third, we did not examine the blood levels of many other factors related to obesity and diet because of limited amounts of serum. Finally, the significance of the marginal, non-significant changes observed in this study can be clarified by using a larger sample size. Future studies that can overcome these limitations are required to validate the present findings.

## Conclusion

Daisaikoto prevents obesity and post-dieting weight regain by reversing obesity-induced dysbiosis and reducing serum corticosterone. Although daisaikoto inhibits fat absorption, it does not induce excessive weight loss, and has no apparent harmful side effects. Thus, daisaikoto may represent a promising, safe, and effective anti-obesity drug candidate.

## Data Availability Statement

The data generated in this study can be accessed in the BioProject database (accession: PRJDB8700).

## Ethics Statement

The animal study was reviewed and approved by Experimental Animal Care Committee of the Kracie Pharma (Toyama, Japan).

## Author Contributions

TK designed the study and wrote the manuscript. TK and MO performed the experiments and analyzed the data. NF and RT revised the manuscript. All authors discussed the results and contributed to the final manuscript.

## Conflict of Interest

All authors are employees of Kracie Pharma, Toyama, Japan.

## Footnotes

1 http://www.qiime.org

2 https://rdp.cme.msu.edu/classifier/classifier.jsp

## Supplementary Material

FIGURE S1 Expression analysis of mRNAs associated with obesity. Data represent the mean ± SEM (*n* = 5). ^#^*p* < 0.05, ^##^*p* < 0.01 vs. normal chow; ^∗^*p* < 0.05, ^∗∗^*p* < 0.01 vs. cycle-HFD, as determined by Tukey’s test.

TABLE S1 Expression analysis of mRNAs associated with obesity.
